# Antifungal Activities of *Bacillus subtilis* Lipopeptides to Two *Venturia inaequalis* Strains Possessing Different Tebuconazole Sensitivity

**DOI:** 10.3389/fmicb.2019.02327

**Published:** 2019-10-22

**Authors:** Hélène Desmyttere, Caroline Deweer, Jérôme Muchembled, Karin Sahmer, Justine Jacquin, François Coutte, Philippe Jacques

**Affiliations:** ^1^Univ. Lille, INRA, ISA-Yncréa, Univ. Artois, Univ. Littoral Côte d’Opale, EA 7394 – ICV – Institut Charles Viollette, Lille, France; ^2^Civil and Geo-Environmental Engineering Laboratory (LGCgE), ISA – Yncréa, Lille, France; ^3^MiPI, TERRA Teaching and Research Centre, Gembloux Agro-Bio Tech, University of Liège, Gembloux, Belgium

**Keywords:** *V. inaequalis*, *Bacillus subtilis*, lipopeptides, biofungicide, antifungal activity

## Abstract

Within the framework of biocontrol development, three natural substances produced by *Bacillus subtilis*, called lipopeptides, have been studied: fengycin (F), surfactin (S), and mycosubtilin (M). Their antifungal properties were tested *in vitro*, in liquid medium, on two strains of *Venturia inaequalis*, ascomycete fungi causing apple scab. These two strains were, respectively sensitive and less sensitive to tebuconazole, an active substance of the triazole family. These three molecules were tested on their own, in binary (FS, FM, SM) and ternary mixtures (FSM). The antifungal activities of lipopeptides were estimated by calculating an IC_50_, compared to tebuconazole chemical substance. In tests involving the sensitive strain, all lipopeptide modalities exhibited antifungal activity. However, modalities involving fengycin and its mixtures exhibited the best antifungal activities; the activity of fengycin alone being very similar to that of tebuconazole. Interestingly, regarding the strain with reduced sensitivity to tebuconazole, surfactin and fengycin alone were not efficient while mycosubtilin and the different mixtures showed interesting antifungal activities. Specifically, the antifungal activity of FS and FSM mixture were equivalent to that of tebuconazole. For both fungal strains, microscopic observations revealed important morphological modifications in the presence of fengycin and in a less important proportion in the presence of surfactin but not in the presence of mycosubtilin. Overall, this study highlights the diversity in mode of action of lipopeptides on apple scab strains.

## Introduction

Apple scab, also known as black spot, is the foremost disease affecting commercial apple orchards worldwide (Bowen et al., 2011). This disease is among the most economically important ones, being accountable for huge crop losses, with up to 70% reduction in apple production (MacHardy, 1996). The pathogen responsible for apple scab is a hemibiotrophic ascomycete fungus, called *Venturia inaequalis* Cooke (Winter) for the teleomorph form and *Spilocaea pomi* Fries (or *Fusicladium pomi*) for the anamorph. *V. inaequalis* attacks leaves, flowers and fruits, causing visible lesions, especially at the early stages of plant development, when they are the most sensitive (Bowen et al., 2011). Consequently, yield reductions result directly from unmarketable infected fruits, and indirectly from repeated defoliation. Although apple scab can be managed through integrated practices like genetic and prophylactic methods, the use of fungicides remains the main practice with up to 15–20 treatments per year (Parisi et al., 2004; Brun et al., 2008; Carisse and Jobin, 2012). It has been estimated that triazoles are the most used class of fungicides, accounting for 20% of fungicide use (Parker et al., 2014). However, the repeated use of single-site chemical fungicide, such as demethylation inhibitor fungicide (DMIs), has rapidly led over time to the development of *V. inaequalis* strains with reduced sensitivity to triazoles fungicides (Köller et al., 1995; Gao et al., 2009; Xu et al., 2010; Villani et al., 2015). Besides, many cases of resistant strains of *V. inaequalis* have been recorded worldwide: in north and south America (Hildebrand et al., 1988; Köller et al., 1991, 1995; Braun and McRae, 1992; Carisse and Pelletier, 1994; Mondino et al., 2015), in Europa (Kunz et al., 1997; Gao et al., 2009; Xu et al., 2010) and in Asia (Shirane et al., 1996; Vijaya Palani and Lalithakumari, 1999). In organic farming, apple scab can be managed using sulfur or copper. However, the heavy use of copper can lead to significant environmental problems (European Food Safety Authority [EFSA], 2013).

Therefore, there is an increasing need for new safe and environmental-friendly alternatives, such as biopesticides. These are defined as living organisms or products derived from them, which present an antagonistic activity against a targeted pest. Members of the *Bacillus* species, for instance, are known for producing a wide variety of antimicrobial compounds (Ongena and Jacques, 2008). In particular, the rhizobacterium *Bacillus subtilis* is one of the most commonly used and well-studied organism (Caulier et al., 2019). With an average of about 4–5% of its genome dedicated to secondary metabolites synthesis, it has the potential to produce more than two dozen structurally diverse antimicrobial compounds (Stein, 2005). Henceforth, some *B. subtilis* strains have already been registered and commercialized as biopesticides, such as SERENADE^®^ (*Bacillus subtilis* str. QST 713) to control plant pathogens (Falardeau et al., 2013).

**FIGURE 1:**
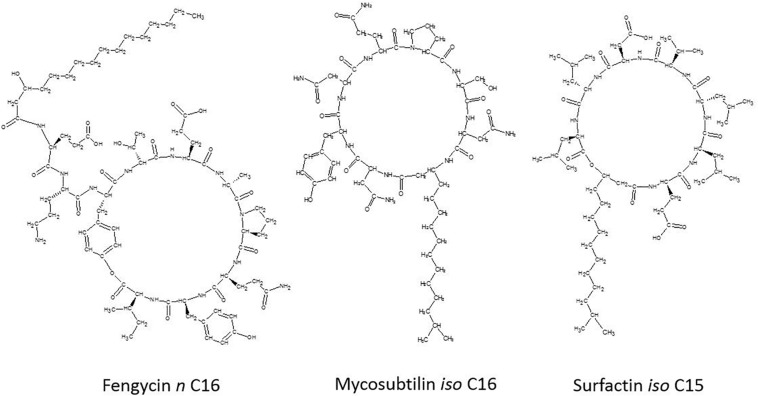
General representation of the chemical structure of lipopeptides from each family: fengycin *n* C16, mycosubtilin *iso* C16, and surfactin *iso* C15.

The lipopeptides produced by *B. subtilis* or *Bacillus velezensis* are considered as the main compounds involved in its biocontrol effect (Ongena and Jacques, 2008). *B. subtilis* lipopeptides are amphiphilic secondary metabolites produced by non-ribosomal peptide synthetases (NRPSs), composed of a cyclic peptide moiety (hydrophilic) linked to a fatty acid chain (hydrophobic) (Stein, 2005; Jacques, 2011; Cochrane and Vederas, 2014). According to their amino acid sequence, cyclic lipopeptides (CLPs) are classified in three distinct families, as shown in Figure 1: fengycin (fengycin and plipastatin), iturin (iturin, mycosubtilin, bacillomycin, and mojavensin), and surfactin (lichenysin and pumilacidin) (Ongena and Jacques, 2008; Jacques, 2011). Members of the fengycin family are decapeptides with a β-hydroxy fatty acid chain (C13–C19) (Hamley et al., 2013; Caulier et al., 2019). Interest in fengycin arises from its strong antifungal activity, specifically against filamentous fungi (Deleu et al., 2008). Members of the iturin family are heptapeptides with a β-amino fatty acid (C14–C18), displaying limited antiviral but strong antiyeast and antifungal activity (Maget-Dana and Peypoux, 1994). Surfactins are heptapeptides containing a β-hydroxy fatty acid tail (C12–C16) (Hamley et al., 2013; Caulier et al., 2019). Surfactin are mostly known for their powerful biosurfactant properties (Ongena and Jacques, 2008; Jacques, 2011), but are generally considered to have limited fungitoxicity (Pérez-Garcia et al., 2011). Synergistic activity of these lipopeptides have been already demonstrated for surfactin and iturin, surfactin and fengycin and iturin and fengycin (Ongena and Jacques, 2008).

*In vitro*, the antagonism of a variety of *B. subtilis* strains, or their cell-free supernatant (followed by lipopeptide identification) as well as the direct activity of isolated lipopeptides have been assessed on a broad range of pathogenic fungi (Touré et al., 2004; Leclère et al., 2005; Romero et al., 2007a; Kim et al., 2010; Dunlap et al., 2011; Tao et al., 2011; Liu et al., 2014; Arroyave-Toro et al., 2017; Mejri et al., 2017; Mihalache et al., 2017). A novel approach is to consider these microbial compounds as future biocontrol agent. Purified molecules have been thus tested *in vivo* on major pathogens such as *Botrytis cinerea* (Farace et al., 2015), *Zymoseptoria tritici* (Mejri et al., 2017), and *Fusarium oxysporum* (Mihalache et al., 2017).

Therefore, as natural substances of microbial origin, lipopeptides represent a promising biocontrol agent, which has been increasingly studied in recent years. The aim of this study is to exhaustively assess, for the first time, the potential antifungal properties of fengycin, mycosubtilin, and surfactin CLPs of *B. subtilis* lipopeptides against the causal agent of apple scab.

## Materials and Methods

### Culture Conditions and Inoculum Preparation

The two *V. inaequalis* strains were provided by IRHS ECOFUN team, INRA Angers-Nantes centre (France). These two strains were distinguished by Muchembled et al. (2018) being respectively sensitive to tebuconazole (S755) and with reduced sensitivity to tebuconazole (rs552). Both strains were maintained on malt agar medium at 20°C, in the dark. Spores were collected from 20 days old cultures in glucose peptone (1.43% glucose and 0.71% bactopeptone). Spore suspensions were calibrated at 5 × 10^4^ spores ml^–1^.

### Lipopeptide Production

Lipopeptides used in this study were produced in shaked flasks using modified Landy media according to Coutte et al. (2010a) for surfactin and fengycin production and Béchet et al. (2013) for mycosubtilin production. They were purified from the fermentation broth using two steps ultrafiltration methods on 10 kDa membrane including four steps of diafiltration as previously described by Coutte et al. (2010b) and Jauregi et al. (2013). After freeze drying, the obtained powders of lipopeptides were then characterized using RP-HPLC and HPLC-MS as previously described (Mejri et al., 2017). In brief, surfactin are composed of isoforms with fatty acid chain of C12–C16, mycosubtilin with fatty acid chain of C15–C18 and fengycin with saturated and unsaturated fatty acid chain of C14–C18.

These three lipopeptides were tested alone (F, M, S), in binary (FS, FM, SM) and ternary mixtures (FSM) (Table 1). The antifungal activities of lipopeptides were compared to the active substance of reference: tebuconazole (Sigma-Aldrich, St. Louis, United States) which is a penetrating and systemic fungicide, from the triazoles family (DMIs).

**TABLE 1:** Lipopeptides used in this study and their *Bacillus subtilis* producing strains.

**Lipopeptide(s)**	**Code**	***B. subtilis* strain**	**References**
Fengycin	F	Bs2504	Ongena et al. (2007)
Mycosubtilin	M	BBG125	Béchet et al. (2013)
Surfactin	S	BBG131	Coutte et al. (2010a)
Fengycin + Mycosubtilin	FM	Mix (50:50 w/w)	
Fengycin + Surfactin	FS	Mix (50:50 w/w)	
Surfactin + Mycosubtilin	SM	Mix (50:50 w/w)	
Fengycin + Surfactin + Mycosubtilin	FSM	Mix (33:33:33 w/w/w)	

### *In vitro* Assay

Direct activity of lipopeptides on *V. inaequalis* was tested in liquid medium within microplates (Muchembled et al., 2018). Sterile flat-bottomed polystyrene 96-well plates were used (Corning^®^ Costar^®^ 3595). Lipopeptides or tebuconazole powders were solubilized in dimethyl sulfoxide (DMSO), and mixed in glucose peptone (1.43% glucose and 0.71% bactopeptone), in order to get a final 0.1% v/v of DMSO. Briefly, for each of the eight treatments (F, M, S, FM, FS, MS, FSM, T), a range of eight concentrations was made (Table 2), and 140 μl per well was distributed in microplates, one concentration per line. Thereafter, 60 μl of a calibrated spore suspension was added to eight wells per line (for a final volume of 200 μl of medium), which corresponds to eight replicates per concentration. The first four columns were left free from spore suspension (60 μl glucose peptone added instead) and used as a control to measure the net optical density (OD). The microplates were then sealed and left agitating (140 rpm) for 6 days at 20°C, in the dark.

**TABLE 2:** Concentrations of the different products used in the microplates experiment.

**Product**	**Concentrations (mg l**^–^**^1^)**							
Lipopeptides	0	0.0244	0.0977	0.3906	1.5625	6.25	25	100
Tebuconazole	0	0.0152	0.0533	0.1866	0.6531	2.2857	8	28

### Data Analysis

On the sixth day, the OD values were read at 635 nm with a microplate reader (Biotek EL 808; Sharma et al., 2004), in order to determine the IC_50_ (concentration of the substance to which 50% of the fungal development is inhibited), using a non-linear regression (logistic model). The experiment was repeated four times independently, to manage the intra- and inter-experiments variability. Differences between modalities (treatments × strains) were tested with an *F*-test comparing a non-linear regression model with distinction between modalities, to a model without this distinction (Ritz and Streibig, 2008). Different *F*-tests were performed. Some *F*-test compared lipopeptide treatments for each strain independently: firstly without tebuconazole to compare lipopeptides among each other, and secondly with tebuconazole, to compare lipopeptide activity to that of tebuconazole. Other *F*-tests compared results of both strains for each modality. Calculated from four independent repetition, each IC_50_ value is supported by its 95% confidence interval. Statistical analyses were performed using R-software (R Core Team, 2016. R: A language and environment for statistical computing. R Foundation for Statistical Computing, Vienna, Austria)^1^.

### Optical Microscopy

Right after microplates reading, *V. inaequalis* was observed under an optical microscope in order to check the fungus morphology (Nikon Eclipse 80i, with Nikon Digital Camera Ddxm1200c). Three samples of each single compound (F, M, S, and tebuconazole) were taken from microwells at IC_50_ concentration and were compared to untreated control. First, a 5 μl drop of lactophenol blue solution was placed on glass slides. Then, 5 μl of sample (mycelium fragment in glucose peptone from microwells) were added before applying the coverslip.

## Results

### Effect of Lipopeptides on *V. inaequalis* Growth

**FIGURE 2:**
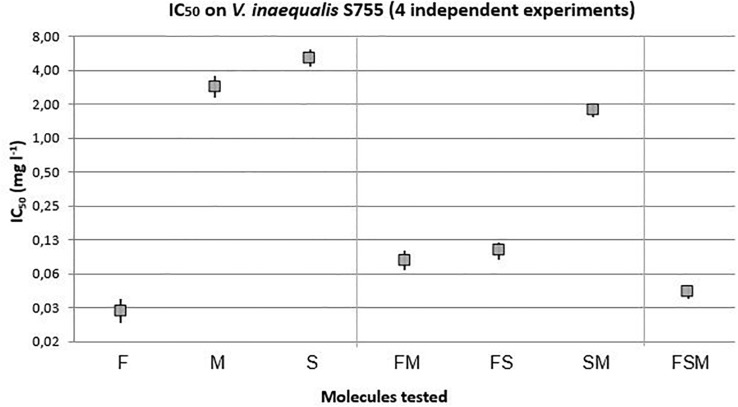
IC_50_ with 95% confidence interval of lipopeptides modalities on the sensitive strain of *V. inaequalis* to tebuconazole (*F* = 256.77, 6 and 1595 df, *p*-value < 0.0001).

The effect of the different samples of lipopeptides were first tested on the tebucanozole sensitive strain, S755 (Figure 2). The fengycin with an IC_50_ of 0.03 mg l^–1^ ([0.02–0.04]) was the lipopeptide with the most remarkable antifungal activity among the three lipopeptides tested alone. With respectively 2.84 mg l^–1^ ([2.23–3.56]) and 5.15 mg l^–1^ ([4.28–6.18]), mycosubtilin and surfactin were the lipopeptides with lower antifungal activities than fengycin. Among the mixtures, the combination of the three lipopeptides was more effective than fengycin + mycosubtilin, and fengycin + surfactin, while surfactin + mycosubtilin being the least effective. However, this surfactin + mycosubtilin binary mixture is significantly more effective than mycosubtilin or surfactin alone. It can be noted that the three lipopeptide mixture (FSM, 0.045 mg l^–1^ [0.04–0.05]) was almost as effective as fengycin alone (0.03 mg l^–1^ [0.02–0.04]).

**FIGURE 3:**
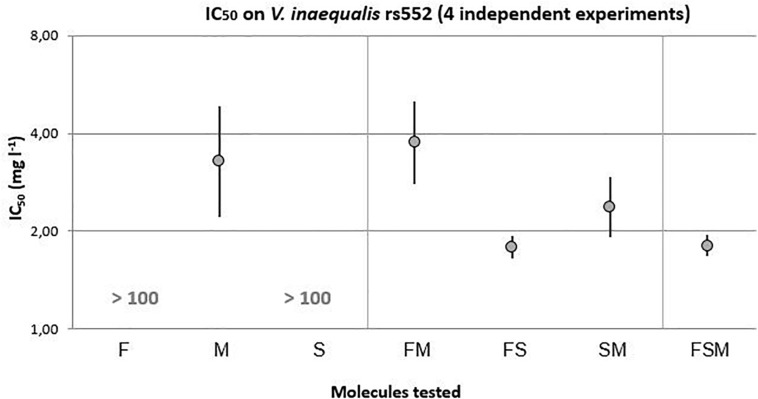
IC_50_ with 95% confidence interval of lipopeptides modalities on the *V. inaequalis* strain with reduced sensitivity to tebuconazole (*F* = 24.559, 4 and 1210 df, *p*-value < 0.0001).

On the tebuconazole reduced sensitivity strain, rs552 (Figure 3), mycosubtilin with an IC_50_ of 3.28 mg.l^–1^ [2.21–4.86] was the only lipopeptide with an important antifungal activity in comparison to fengycin and surfactin. In the light of the 95% confidence interval, similar levels of antifungal activity were obtained with M-containing binary mixtures: SM (IC_50_ = 2.37 mg l^–1^ [1.92–2.93]) and FM (IC_50_ = 3.75 mg l^–1^[2.79–5.04]). Even though the fengycin and surfactin alone did not show any antifungal activity, their combination (FS) have showed a higher antifungal activity than mycosubtilin with an IC_50_ of 1.79 mg l^–1^ [1.65–1.94]. A synergistic effect between fengycin and surfactin might therefore be occurring. Moreover, the ternary mixture FSM (1.81 mg l^–1^ [1.67–1.95]) showed an equivalent antifungal activity to FS.

*F*-tests were carried out for each modality allowing comparison between strains (Table 3). While fengycin and surfactin did not have any effect on the rs552 strain, mycosubtilin revealed a very close antifungal activity on both strains (2.32 mg l^–1^ [1.96–2.74] for S755 and, 3.34 mg l^–1^ [2.62–4.25] for rs552). Moreover, every lipopeptides mixtures (FM, FS, SM, and FSM) were always significantly more effective on the sensitive strain S755. The IC_50_ of tebuconazole was statistically lower for S755 (0.02 mg l^–1^ [0.02–0.03]), than for rs552 (1.65 mg l^–1^ [1.50–1.82]), being less sensitive.

**TABLE 3:** Comparison of all modalities IC_50_ with 95% confidence interval between strains with different sensitivity to tebuconazole (four independent experiments).

			**S75 5 strain**		**rs552 strain**	
	***F* value**	***p*-value**	**IC_50_ (mg L^–1^)**	**Confidence interval (95%)**	**IC_50_ (mg L^–1^)**	**Confidence interval (95%)**
F	Non-calculable		0.033	[0.025–0.043]	>100	
M	6.918 (1 and 492 df)	0.0087	2.315	[1.955–2.740]	3.339	[2.623–4.252]
S	Non-calculable		5.984	[4.188–8.551]	>100	
FM	401.02 (1 and 487 df)	<0.0001	0.079	[0.062–0.100]	3.21	[2.656–3.879]
FS	227.57 (1 and 486 df)	<0.0001	0.102	[0.084–0.123]	2.191	[1.876–2.559]
SM	23.445 (1 and 440 df)	<0.0001	1.756	[1.605–1.922]	2.647	[2.182–3.213]
FSM	360.56 (1 and 425 df)	<0.0001	0.043	[0.035–0.054]	2.085	[1.823–2.385]
Tebuconazole	862.15 (1 and 450 df)	<0.0001	0.022	[0.019–0.025]	1.65	[1.499–1.815]

Moreover, two other *F*-tests were run for each strain to compare all modalities with tebuconazole as a reference (data shown in Supplementary Material). On the sensitive strain (*F* = 305.67, 7, and 1804 df, *p*-value < 0.0001), it can be outlined that fengycin (0.03 mg l^–1^ [0.02–0.04]), exhibited a strong antifungal activity at the same level as tebuconazole (0.02 mg l^–1^ [0.02–0.03]). Likewise, on the strain with reduced sensitivity (*F* = 21.97, 5 and 1452 df, *p*-value < 0.0001), the most important antifungal activities were FS (1.8 mg l^–1^ [1.67–1.94]) and FSM (1.82 mg l^–1^ [1.7–1.95]) that showed an IC_50_ as low as tebuconazole (1.86 mg l^–1^ [1.68–2.05])

### Effect of Lipopeptides on *V. inaequalis* Morphology

**FIGURE 4:**
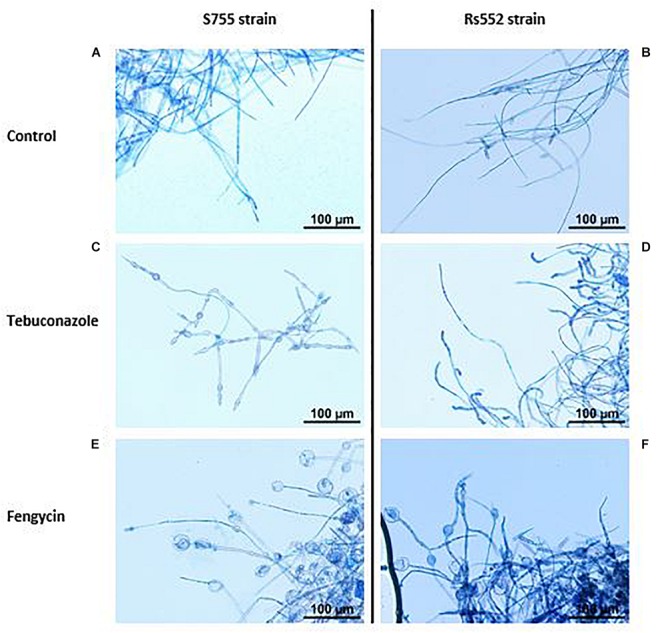
Effect of tebuconazole and fengycin on the morphology of *V. inaequalis* mycelium. Optical microscopy photos were taken on the sixth day after microplate’s inoculation with calibrated spores’ suspension, with lactophenol blue staining. **(A)** S755 control; **(B)** rs552 control; **(C)** S755 treated with tebuconazole (0.65 mg l^–1^); **(D)** rs552 treated with tebuconazole (0.65 mg l^–1^); **(E)** S755 treated with fengycin (0.39 mg l^–1^); **(F)** rs552 treated with fengycin (0.39 mg l^–1^).

The untreated mycelium of *V. inaequalis* has first been observed under photonic microscopy, in order to confirm the regular morphology in the absence of CLPs (Figure 4). For both strains, mycelium appeared to be well developed, with long hyphae in the control (Figures 4A,B). *V. inaequalis* has also been observed under tebuconazole treatment. On the sensitive strain, oval-shaped swollen structures, of small sizes, were observed along the mycelium, with a concentration-dependent effect (Figure 4C). However, on the strain with reduced sensitivity, no such morphological changes were noticed. The mycelium looked well developed, although looking slightly more branched and thicken on hyphae’s tips (Figure 4D). In the presence of fengycin, original swollen structures were observed, mainly at the tip of hyphae (Figures 4E,F). Interestingly, these vesicle-like structures seemed of varied type: from slightly swollen cells to large clear bag-like structures, which even looked torn at times. These modifications were observed on both strains, but were less ubiquitous on rs552. These structures were different from the kind observed under tebuconazole treatment. Although the presence of vesicle-like structures is systematic in the presence of fengycin, these same structures can be observed in a less systematic way in the presence of surfactin. On the contrary, in the case of mycosubtilin, no vesicle-like structures were observed on both strains. Therefore, the mycelium in the presence of mycosubtilin had a morphology identical to that of the control.

## Discussion

### Lipopeptide Have a Good Efficacy Against *V. inaequali*s

Our results revealed, for the first time, the existence of *in vitro* antifungal properties of CLPs from *B. subtilis* on *V. inaequalis*, and in some cases, the concentration range was comparable with the chemical reference tebuconazole.

Even though surfactin is generally considered as having little or no antifungal properties on its own (Ongena and Jacques, 2008; Gonzalez-Jaramillo et al., 2017), we highlighted a clear antifungal activity against S755 strain like other studies (Inès and Dhouha, 2015; Krishnan et al., 2019). Mycosubtilin inhibited both strains, with a statistically similar antifungal activity (IC_50_ = 2.32 mg l^–1^ [1.96–2.74] on S755 and 3.34 mg l^–1^ [2.62–4.25 mg l^–1^] on rs552). Mycosubtilin has already been showed to inhibit several phytopathogenic fungi (Leclère et al., 2005; Mejri et al., 2017). Comparing our IC_50_ results to other studies is not straightforward, since most of them evaluated the CLPs antifungal efficacy by using inhibition or growth rate/percentage (Touré et al., 2004; Leclère et al., 2005; Romero et al., 2007b; Dunlap et al., 2011; Tao et al., 2011; Liu et al., 2014; Arroyave-Toro et al., 2017; Mihalache et al., 2017). However, in a recent work of Mejri et al. (2017), the IC_50_ of mycosubtilin, in liquid medium, was evaluated at 1.4 mg l^–1^ on *Z. tritici*, which is close to our results. In this study on *Z. tritici*, IC_50_ were above 100 mg l^–1^ for fengycin and surfactin treatments (Mejri et al., 2017), like our results on rs552. Interestingly, this study was conducted on the T01193 strain, which also has reduced sensitivity to several DMIs (S. Mejri, personal communication).

Furthermore, little is known about lipopeptides’ activity while in mixtures, although they could act in a synergistic manner (Ongena and Jacques, 2008). In our experiments, better efficacies were obtained with SM mixtures on S755 than lipopeptides on their own. On rs552, the M-containing binary mixtures (FM and SM) were relatively as effective as mycosubtilin alone. Also, a very strong synergistic effect was observed with the mixture FS transforming two non-inhibitory molecules in an efficient complex. Synergistic activities have already been showed on phytopathogenic fungi, with the mixture surfactins + mycosubtilins or surfactins + iturins (Maget-Dana et al., 1992; Romero et al., 2007b; Deravel et al., 2014; Mejri et al., 2017; Mihalache et al., 2017). It is also known that anionic and non-ionic biosurfactants as for example surfactins and iturins can form mixed micelles, acting more efficiently than simple micelles on biological membranes (Maget-Dana et al., 1992; Muñoz et al., 2004; Jauregi et al., 2013). However, less information are available about the potential synergistic effect of fengycin and surfactin which are both anionic biosurfactants.

### Lipopeptides Differently Induce Fungal Morphological Modifications

Through our microscopic observations, we noticed consistent morphological modifications on *V. inaequalis* under fengycin treatments. Many vesicle-like swollen structures were observed on both strains. Morphological damages by *B. subtilis* lipopeptides have already been noticed on other fungi. However, this is the first time such swollen structures on mycelium are well characterized in the presence of isolated fengycin. Under scanning electron microscopy (SEM), Romero et al. (2007a) observed a loss of turgidity of *Podosphaera fusca* conidia following treatment with a cell-free supernatant of *B. subtilis*. Increase vacuolization and disorganization of the cytoplasm, resulting from membrane disruption by small vesicle-like structures, was visualized with transmission electron microscopy (TEM) (Romero et al., 2007a). Mihalache et al. (2017) also spotted deeply folded, shrunken, wrinkled and partially distorted hyphae, as well as subterminal vesicles and intercalary swelling on *F. oxysporum* mycelium, under M or SM treatments, using SEM. More recent data, from Park et al. (2019), have shown vesicle-like structures with surfactin (from *B. velenzenis*) on *Colletotrichum gloeosporioides*. The surface of the mycelium was rugged and unusually swollen and rough, suggesting the peptide affects directly the fungal cell wall components (chitin, glucans, and glycoproteins) to exhibit antifungal activity (Park et al., 2019).

Clear disruptions were observed after linking antifungal activity data with morphological microscopic observations. Indeed, on S755, for the same level of antifungal activity, we did not observe the same type of morphological modifications according to treatments: large vesicle-like structures could be seen in the presence of fengycin, while small oval-shaped swollen structures, of small sizes, could be observed with tebuconazole. Moreover, in the case of fengycin, the same kind of vesicle-like structures were observed on both strains even though fengycin had a strong activity on S755 but did not show any activity on rs552. The same goes for surfactin-treated fungi, where similar vesicle-like structures were seen on both strains, irrespective of the activity. A work by Vanittanakom et al. (1986) also relates for the first time morphological changes in different fungi (bulging, curling, emptying) but this does not seem directly related to antifungal activity. This is in agreement with our results, for which the presence of original vesicle-like structures does not seem correlated with antifungal activity. Regarding mycosubtilin, we never observed vesicles (neither on S755, nor on rs552), even if there was always antifungal activity for both strains.

### Lipopeptides Differently Inhibit Tebuconazole Sensitive and Less Sensitive *V. inaequalis* Strains

Another originality of our approach is to compare, for the first time, CLPs activity on fungal strains possessing different sensitivity to fungicides. Our tests clearly confirmed the difference in tebuconazole sensitivity between both 755 and 552 (Muchembled et al., 2018). Tebuconazole is an inhibitor of sterol biosynthesis (DMIs), which specifically targets the 14α-demethylase (or CYP51), an important regulatory enzyme in the ergosterol biosynthetic pathway (Yoshida and Aoyama, 1987). Inhibition of this enzyme disrupts ergosterol synthesis, leading to the reduction of ergosterol content and thus the formation of cell membranes with altered structure and functions, such as fluidity and permeability (Leroux et al., 2008; Price et al., 2015). Three major mechanisms may be implicated in DMI-resistance (Cools et al., 2013; Price et al., 2015). The first is the alteration of the target enzyme, which results in reduced affinity to fungicides (Leroux et al., 2008; Cools et al., 2013; Parker et al., 2014). The second mechanism involves the overexpression of CYP51 (Schnabel and Jones, 2001; Pfeufer and Ngugi, 2012; Villani et al., 2016) while the third mechanism is an efflux phenomenon, enabling a decrease of the intracellular drug accumulation, attributed to overexpression of membrane transporter proteins involved in multidrug resistance (MDR) (Leroux et al., 2008; Cools et al., 2013). Two efflux systems are involved in MDR: the ATP-Binding Cassette (ABC), which utilizes energy from ATP hydrolysis, and the Major Facilitators Superfamily (MFS) transporters, which employ the proton motive force (Deising et al., 2008).

Moreover, Vijaya Palani and Lalithakumari (1999) found that some DMIs-resistant *V. inaequalis* strains were characterized by significantly reduced total lipid and ergosterol biosynthesis. They postulated that the reduction in ergosterol content could lower membrane permeability, thus hampering the transport of toxicant in the resistant strains. Another study stated that low ergosterol content is linked with increased membrane interaction and destabilization (Falardeau et al., 2013). High fungal ergosterol could buffer fluidity changes, increasing CLPs tolerance (Falardeau et al., 2013).

These modifications of membrane properties and ergosterol concentrations could be correlated with the biological activities of the CLPs on both *V. inaequalis* strains. Previous studies acknowledged that CLPs can interact with biological membranes and especially ergosterol (Pérez-Garcia et al., 2011). However, the mode of action can differ according to the family of CLPs considering that fengycin and surfactin are negatively charged molecules while mycosubtilin is a neutral compound (Falardeau et al., 2013). Polarity appears to be key in these membrane-CLPs interactions depending on the charge of both lipopeptides and membranes (Deleu et al., 2003; Buchoux et al., 2008; Razafindralambo et al., 2009; Juhaniewicz-Dębińska et al., 2019). While iturin is known to possess an ion-conducting pore-forming activity (Maget-Dana and Peypoux, 1994), fengycin and surfactin have concentration-dependent mode of action. At low concentration, fengycin and surfactin induce limited perturbation, such as pore formation (Deleu et al., 2005; Heerklotz and Seelig, 2007; Pérez-Garcia et al., 2011; Cochrane and Vederas, 2014). At higher concentrations, they operate by a detergent mechanism, with complete disruption and solubilization of the lipid bilayer (Deleu et al., 2003, 2005; Ongena and Jacques, 2008; Cochrane and Vederas, 2014). In a study, Patel et al. (2011) suggested the all-or-none leakage/membrane permeabilization effect of fengycin, highlighting crucial interaction with membrane and lipids.

Fluid state of membrane lipids may also be key for effective CLPs insertion (Deleu et al., 2008; Mantil et al., 2019). In agreement, more recent studies, on model membrane, showed that bilayers containing higher levels of ergosterol exhibited an increased tolerance to activity of fengycin at low doses (Mantil et al., 2019). All these information could explain the differences measured in lipopeptide antifungal activity between tebuconazole sensitive and reduced sensitivity strains of *V. inaequalis*.

## Conclusion

Finally, this study revealed a diversity of responses of two strains of *V. inaequalis* exposed to different families of lipopeptides, highlighting the distinct modes of action of each lipopeptide. On both strains, the lipopeptides with the most remarkable antifungal activities (F for S755; FS and FSM for rs552) were very close to the activity of tebuconazole. These antifungal activities obtained *in vitro* present promising results for CLP development as biopesticides. Moreover, according to several ecotoxicity evaluations, CLPs are amongst the least toxic substances and could thus constitute eco-friendly alternatives to chemical pesticides. In the future, we suggest further investigation under *in vivo* and *in situ* experiments, to validate lipopeptides’ efficacy as biocontrol agents in apple orchards.

## Data Availability Statement

The raw data supporting the conclusions of this manuscript will be made available by the authors, without undue reservation, to any qualified researcher.

## Author Contributions

All authors have made direct experimental and/or intellectual contribution to the work, and have read and approved the final version. HD, CD, and JJ have conducted all the *in vitro* experiment with *V. inaequalis.* FC has produced and purified the different lipopeptides from *Bacillus subtilis*. HD, CD, JM, KS, FC, and PJ were principally responsible for analyzing the results and writing the manuscript. CD, JM, FC, and PJ have defined the general design of the experiments. FC and PJ were respectively the scientific coordinator of the AgriBioPOM and ALIBIOTECH funding projects.

## Conflict of Interest

The authors declare that the research was conducted in the absence of any commercial or financial relationships that could be construed as a potential conflict of interest.

## Footnotes

1 https://www.R-project.org/
