# Supplementary material for: Antifungal Activities of Bacillus subtilis Lipopeptides to Two Venturia inaequalis Strains Possessing Different Tebuconazole Sensitivity
Source: Front Microbiol. 2019 Oct 22;10:2327. doi: 10.3389/fmicb.2019.02327 (PMC6817503; doi:10.3389/fmicb.2019.02327)
Supplement: Supplementary file 1 [file Image_1.pdf]

S755 strain 4 manips avec tebuconazole (T)

	S755 Strain	
	IC_{50} (mg l^{-1})	Confidence interval (95%)
F	0.028	[0.023-0.035]
M	2.852	[2.311-3.519]
S	5.017	[4.237-5.942]
FM	0.082	[0.068-0.099]
FS	0.098	[0.084-0.114]
SM	1.807	[1.588-2.055]
FSM	0.043	[0.037-0.05]
T	0.021	[0.018-0.025]
F value	*305.67 (7 and 1804 df)*	
p-value	*< 0.001*	

Specific comparison lipopeptides and tebuconazole (T) for S755 strain

rs552 strain 4 manips avec tebuconazole (T)

	rs552 Strain	
	IC_{50} (mg l^{-1})	Confidence interval (95%)
F	Non calculable	
M	3.247	[2.29-4.604]
S	Not calculable	
FM	3.675	[2.821-4.788]
FS	1.798	[1.667-1.939]
SM	2.423	[2.008-2.923]
FSM	1.82	[1.698-1.951]
T	1.855	[1.679-2.05]
F value	*21.97 (5 and 1452 df)*	
p-value	*< 0.001*	

Specific comparison lipopeptides and tebuconazole (T) for rs552 strain
